# Closure of the Entire Fistula With Highly Effective Chemocauterization Using the Distal Hood Endoscope: A Novel Procedure for the Treatment of Pyriform Sinus Fistula

**DOI:** 10.7759/cureus.59245

**Published:** 2024-04-28

**Authors:** Shuichi Katayama, Takehito Oshio, Kazuhiro Ohtsu, Kiyoshi Sasaki, Yasuo Nakahara, Kosuke Toyooka, Shojiro Hanaki, Kazuyuki Kawamoto

**Affiliations:** 1 Pediatric Surgery, Kurashiki Central Hospital, Kurashiki, JPN; 2 Pediatric Surgery, Shikoku Central Hospital, Ehime, JPN; 3 Pediatric Surgery, Hiroshima Prefectural Hospital, Hiroshima, JPN; 4 Pediatric Surgery, Kochi Health Sciences Center, Kochi, JPN; 5 Pediatric Surgery, National Hospital Organization Okayama Medical Center, Okayama, JPN

**Keywords:** pediatrics, trichloroacetic acid, pyriform sinus fistula, distal hood endoscopy, chemocauterization

## Abstract

Background: Pyriform sinus fistula (PSF) causes a recurrent abscess in the neck. Endoscopic chemocauterization with trichloroacetic acid (TCA) for PSF is a simple, reproducible, and reliable procedure for treating PSF; however, there is concern about complications caused by TCA overflowing into the larynx. To prevent these complications, we devised a highly effective chemocauterization using a distal hooded endoscope (HuDHE). Our aim is to determine the efficacy and safety of HuDHE in children with PSF.

Methods: The main features of HuDHE are as follows (1) an endoscope with a translucent silicon hood at the tip was made; (2) TCA was endoscopically injected into the PSF; and (3) the color change of the mucosa into PSF was endoscopically evaluated. Data on children receiving HuDHE for PSF in the past seven years were collected from medical records.

Results: Data were obtained for eight children receiving HuDHE. The success rate of treatment for PSF after the first TCA chemocauterization was 87.5% (7/8) and the cumulative success rate after the second treatment was 100% (8/8). None of the children had recurrent PSF or serious complications such as vocal cord paralysis after HuDHE.

Conclusion: HuDHE appears to be a less invasive, safe, and effective treatment for PSF.

## Introduction

Pyriform sinus fistula (PSF) is a rare branchial anomaly derived from congenital remnants of the third or fourth branchial cleft [1]. The standard therapy for PSF is complete resection of the fistula. However, significant scarring due to recurrent abscesses or repetitive incisions or drainage increases the risk of developing postoperative complications [1-3]. Although endoscopic chemocauterization with trichloroacetic acid (TCA) is an alternative treatment for PSF, postoperative recurrence has been reported [1]. There were cases reported who had the closed internal orifice of PSF but recurred [2]. A highly effective chemocauterization using a distal hooded endoscope (HuDHE) was designed as a new procedure for PSF treatment to obliterate almost all fistulous tracts. Our aim of this study was to determine the efficacy and safety of HuDHE in children with PSF.

## Materials and methods

PSF is a rare congenital condition. Therefore, following the approval of the Kurashiki Central Hospital Review Board (ethics approval number, #3733), medical records from 2015 to 2021 were retrospectively examined at the five participating hospitals (Kurashiki Central Hospital, Shikoku Central Hospital, Hiroshima Prefectural Hospital, Kochi Health Sciences Center, and National Hospital Organization Okayama Medical Center), and children receiving HuDHE for PSF at each facility were identified.

**Figure 1:**
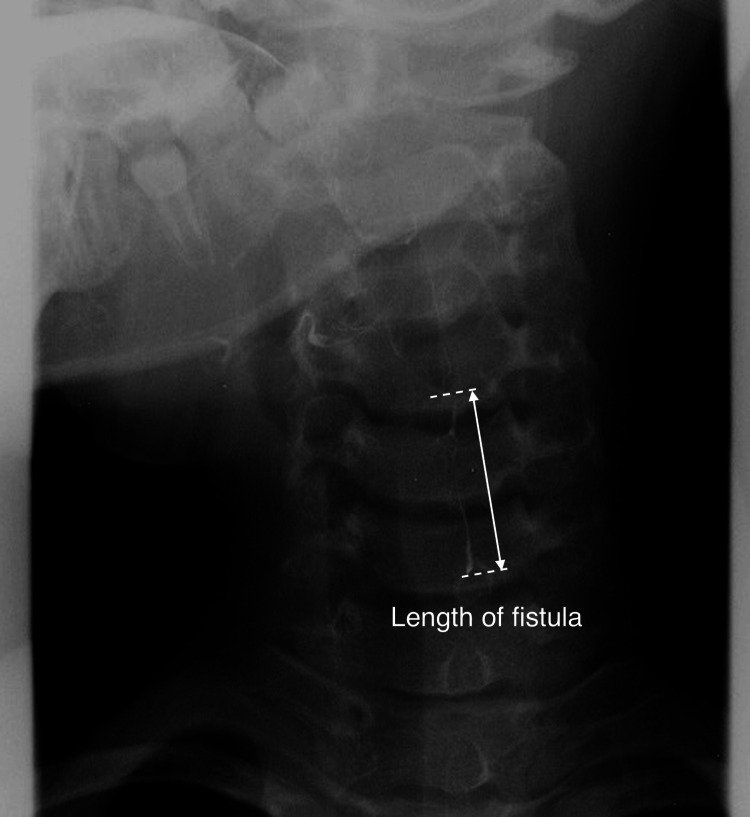
Barium esophagography Barium enters and delineates the pyriform sinus fistula. The length of the fistula was measured in each case.

The study targeted pediatric patients presenting with fever and neck swelling, in whom the fistulous opening in the pyriform fossa was confirmed at each facility. Children where the fistulous opening could not be identified or patients aged 16 years and older were excluded from the study. The medical records of PSF patients were analyzed according to sex, age at onset/surgery, side of the lesion, initial symptoms, length of fistula before operation by esophagography, recurrence, and outcomes (Figure 1).

**Figure 2:**
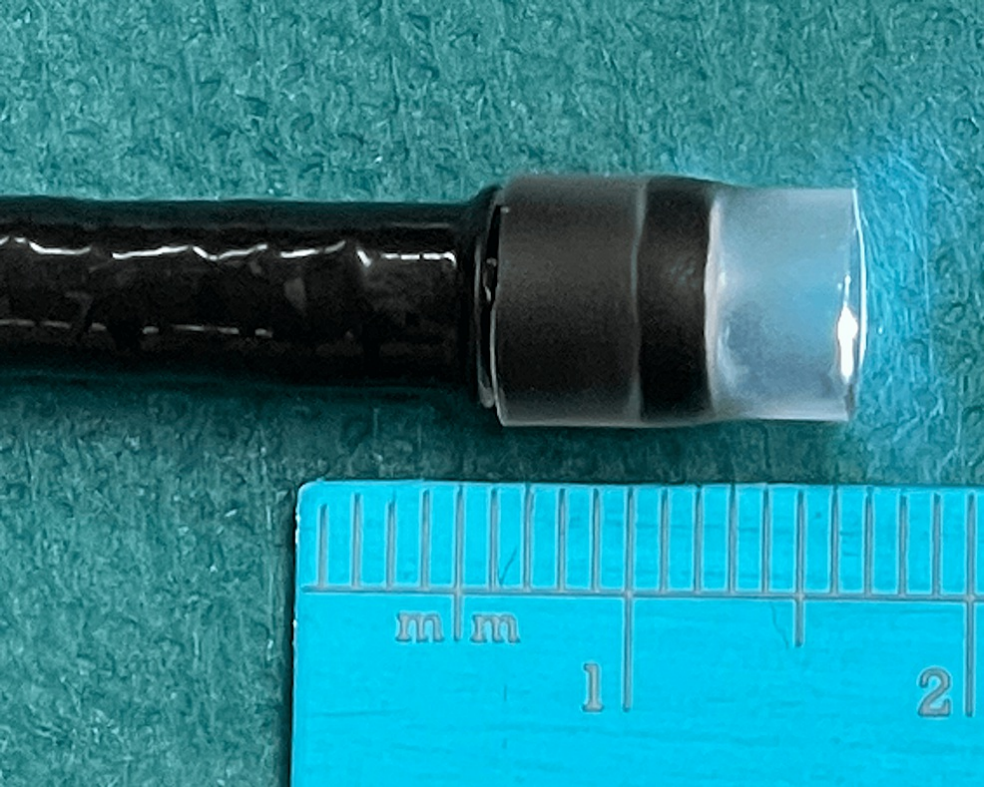
Distal hooded endoscope A hood made from a translucent silicon tube was attached to the tip of the upper gastrointestinal endoscope, bronchoscope, or cholangioscope.

**Figure 3:**
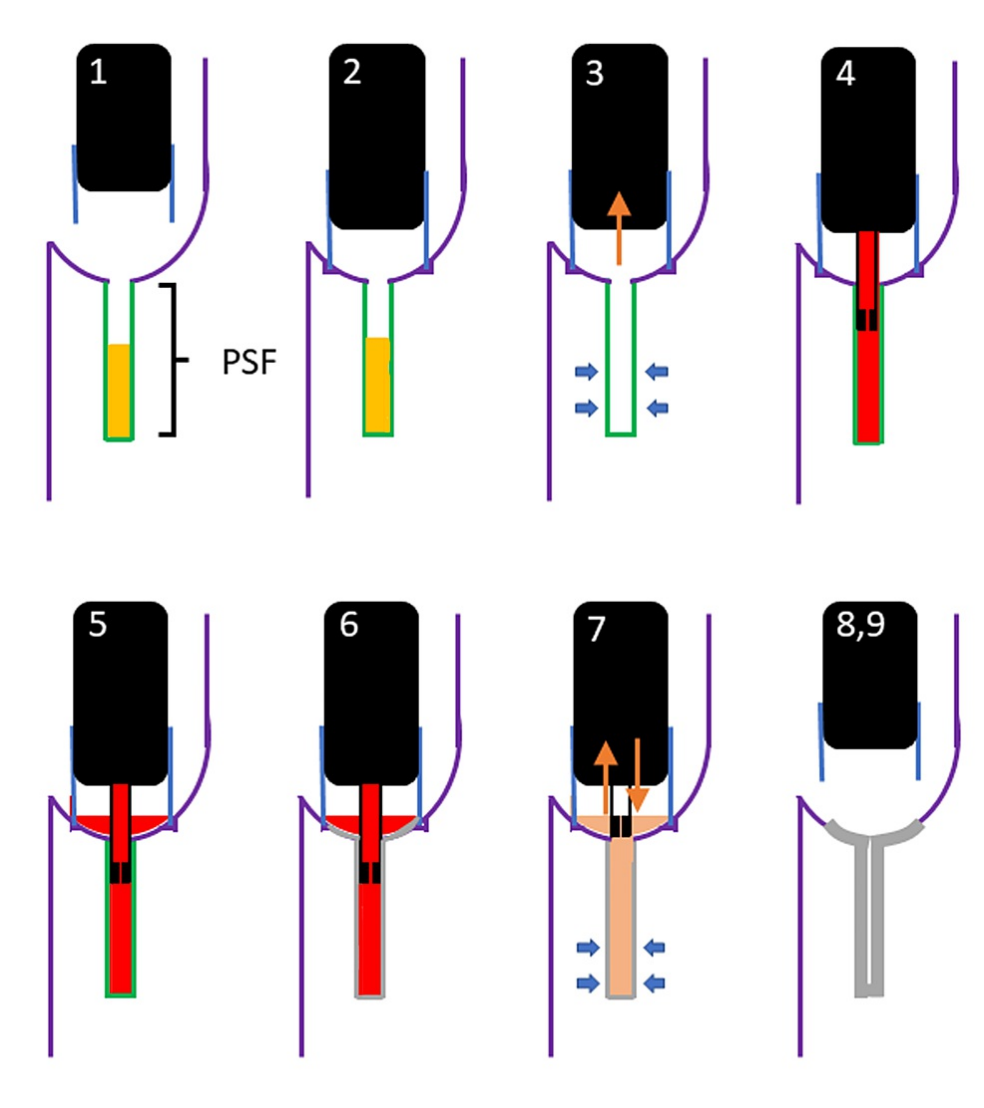
HuDHE 1) Identify PSF; 2) push the hood against the internal orifice of the PSF; 3) compress the anterior neck, and aspirate the PSF; 4) insert the tube into the PSF and inject TCA; 5) observe the TCA overflowing into the hood; 6) confirm whether the mucosa of the PSF is blanched; 7) flush with saline into the PSF; 8) check the color change of the mucosa around the PSF; 9) repeat above steps, if needed. HuDHE: highly effective chemocauterization using a distal hood endoscope; PSF: pyriform sinus fistula; TCA: trichloroacetic acid

The initial examinations to diagnose PSF were barium esophagography and/or enhanced CT scan. Suspension laryngoscopic examination was not performed, and the diagnosis was confirmed by barium esophagography. For the TCA chemocauterizations, patients were placed in the supine position with their ears up and tilted toward the affected side under general anesthesia. A transnasal gastric tube was placed to prevent regurgitation and to identify the esophagus promptly instead of the PSF. The internal orifice of the PSF was identified on the affected side of the pyriform fossa on endoscopy. A translucent silicon hood was attached to the tip of the upper gastrointestinal endoscope, bronchoscope, or cholangioscope that was mainly used in this study, whose diameter was 5.4 mm, 4.8 mm, and 4.9 mm, respectively (Figure 2). TCA was injected through the internal orifice of the PSF using a thin plastic open-tip tube. The tubes have 1.8 mm or 1.9 mm diameter designed for washing and spraying during endoscopy. The description of the procedure is shown in Figure 3.

**Figure 4:**
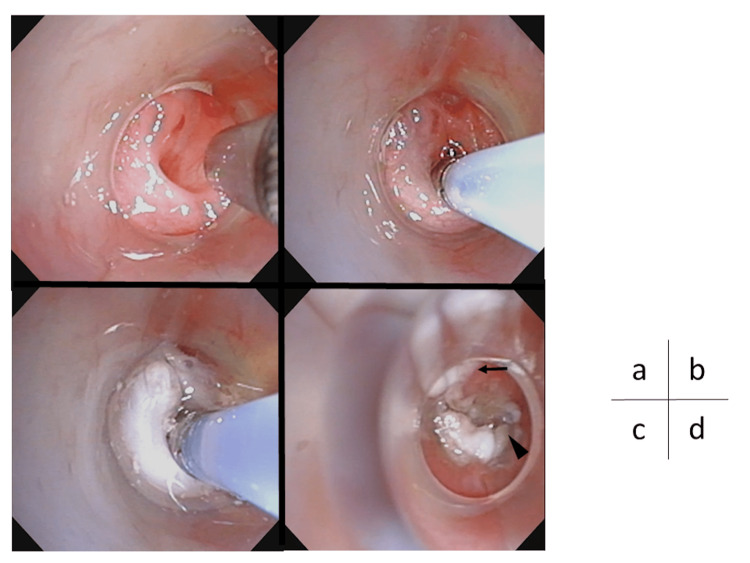
HuDHE in case 6 (a) The internal orifice of the PSF, (b) a tube inserted into the PSF, (c) the color change of mucous of the PSF immediately after TCA injection, (d) TCA exposure (arrows) outside the hood. Arrowhead indicates PSF. HuDHE: highly effective chemocauterization using a distal hood endoscope; PSF: pyriform sinus fistula; TCA: trichloroacetic acid

**Figure 5:**
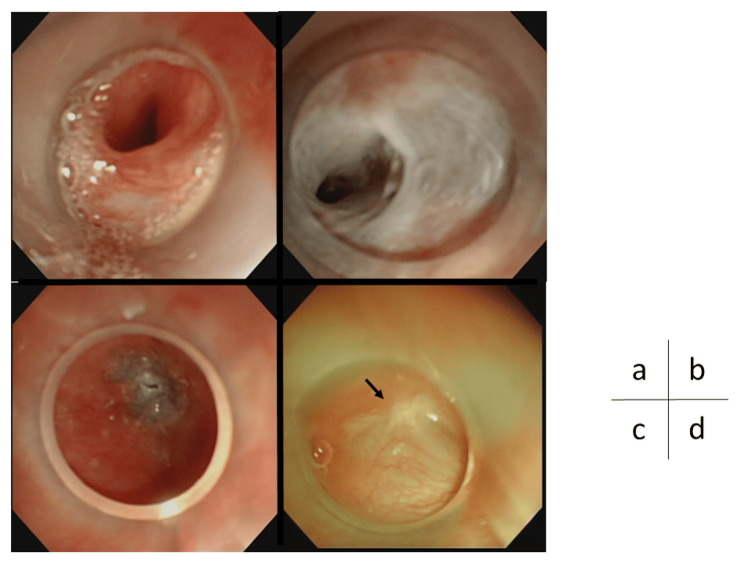
HuDHE in case 5 (a) The internal orifice of the PSF, (b) mucosal degeneration not only at the internal orifice of the PSF but also at the entire fistulous duct, (c) local degeneration around the PSF viewed from the larynx, (d) the PSF closed (arrow) six months after HuDHE. HuDHE: highly effective chemocauterization using a distal hood endoscope; PSF: pyriform sinus fistula

All the detailed procedures are described below. The internal orifice of the PSF is identified (Figures 4a, 5a). A tube is inserted a few millimeters into the internal orifice of the PSF, and TCA is gently injected (Figure 4b). Once TCA overflows outside the PSF, the practitioner waits for 30 to 60 seconds (Figure 4c). Then, PSF should be aspirated and injected with saline alternately with compressing the anterior neck to stir TCA with saline into PSF (Figure 5b). The alteration in coloration of the mucosa surrounding the pyriform sinus is subsequently assessed, and the above steps are repeated if necessary (Figures 4d, 5c).

Oral antibiotics were administered for a few days after HuDHE. Oral feeding was started a few days postoperatively. A follow-up endoscopy was scheduled three to six months later. The second HuDHE was performed if the internal orifice of the PSF was not obliterated yet on the endoscopy. The treatment was terminated when the obliteration of the internal orifice was endoscopically confirmed (Figure 5d).

Statistical analysis

The statistical analysis mainly involved the calculation of percentages. Categorical variables have been expressed in percentages and continuous variables in the median.

## Results

Demographics and symptoms

This study comprised five males and three females. Initial symptoms appeared at a median age of 2.5 years (range, 0 to 8 years). Surgery was performed at a median age of 5.5 years (range, 4 to 8 years). The site was located on the left side for all children. The most common symptom was neck abscesses (seven children, 87.5%) (Table 1).

**Table 1:** Summary of the data of eight children with PSF 1) 10% TCA was used. In the remaining cases, 20% TCA was used. PSF: pyriform sinus fistula; TCA: trichloroacetic acid

Case no.	Age at onset, y	Age at surgery, y	Initial symptom	Total TCA volume, mL	Length of fistula, mL	TCA leakage outside the hood	Complications	Secretion	Follow-up period, y
1	5	6	Neck abscess	0.4	27	–	–	+	7
2-1	4	5	Neck abscess	0.5^1^	20	–	–	–	5
2-2		5		0.2		–	–	–	5
3	0	5	Neck abscess	0.3	17	+	Sore throat	+	4
4	0	6	Neck abscess	0.4	22	–	–	–	4
5	1	4	Neck abscess	0.3	13	–	–	–	2
6	0	5	Neck swelling	0.2	15	+	Sore throat	+	2
7	5	6	Neck abscess	0.5	18	+	–	–	2
8	8	8	Neck abscess	0.4	22	+	Sore throat	–	2

The following treatment approaches were applied to neck abscesses before HuDHE: single incision and four times drainage in one child, a puncture in one, radical open surgery in one, and two times conventional endoscopic TCA chemocauterization in one. All children received antibiotics for neck abscesses. A child with neck swelling due to a giant cyst required intubation for respiratory distress in the neonatal period (case 6). The cyst became smaller without any complication after the Picibanil (OK-432) injection.

Treatment and outcomes

**Figure 6:**
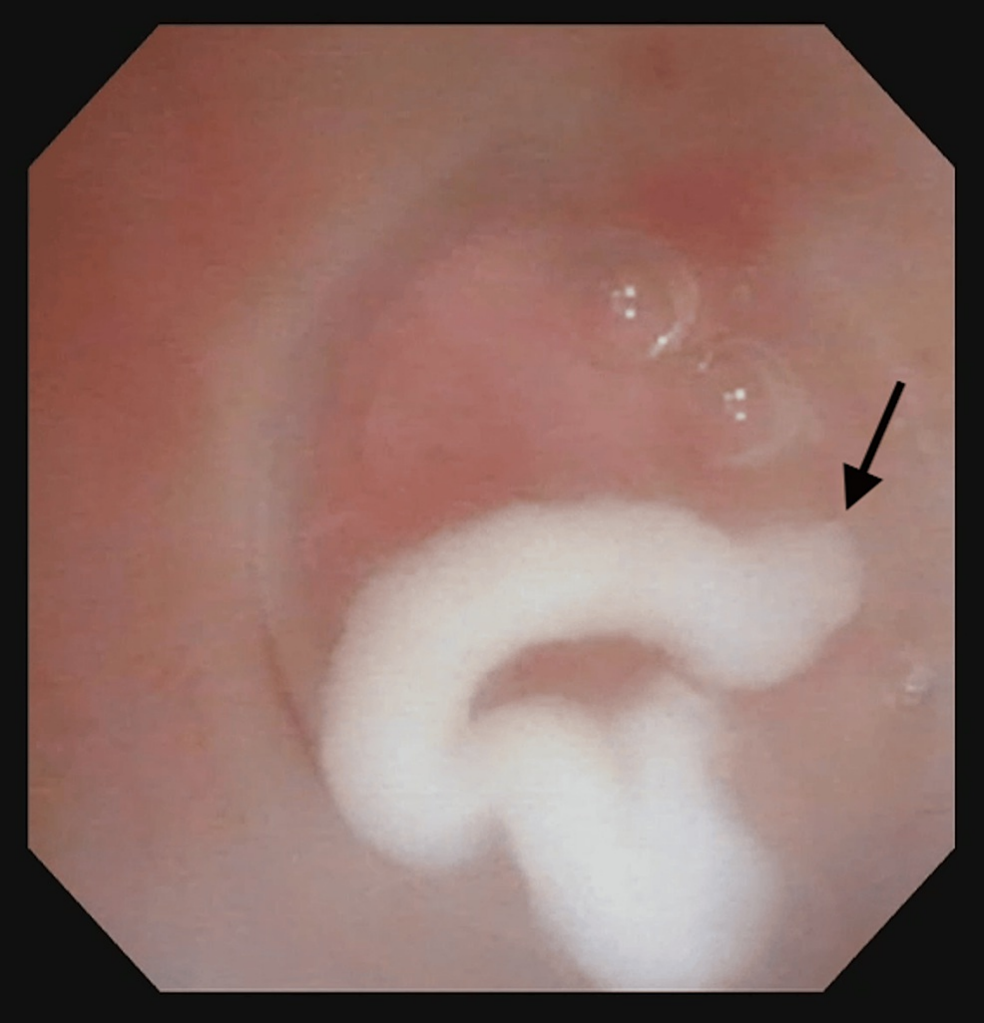
Toothpaste-like secretion in case 6 Toothpaste-like secretion was extruded by compressing the anterior neck around the pyriform sinus fistula (arrow).

All eight patients received HuDHE with TCA when they were between four and eight years old. One child received HuDHE twice (case 2), in whom 10% TCA was used on the first attempt and 20% TCA on the second attempt. In the remaining seven children, only 20% of TCA was used. The success rate of the first HuDHE was 87.5% (7/8 cases), and the cumulative success rate after the second HuDHE was 100%. The median length of the PSF measured by barium esophagography was 19 mm (range, 13 to 27 mm). The mean total volume of TCA was 0.4 mL (range, 0.2 to 0.5 mL). A small amount of TCA leakage outside the hood occurred during HuDHE in four children, in whom the area affected by TCA was from below the left palatine tonsils to the left pyriform sinus (Figure 4d). Transient sore throat as a complication occurred in three of the four children but resolved within a few days. The median length of hospital stay was three days (range, two to four days). In all children, PSF closure was confirmed endoscopically. During a median follow-up period of three years (range, one to six years), none of the children had recurrent PSF or serious complications after HuDHE. The treatments and outcomes are listed in Table 1. All children had no scars on the anterior neck after HuDHE. Interestingly, three of the eight children had toothpaste-like secretion extruded from the PSF during compression and suction (Figure 6). The secretion was not predicted by any imaging modalities before HuDHE.

## Discussion

PSF is a rare congenital anomaly, which is supposed to arise from an incomplete obliteration of the third or fourth branchial pouch during the seventh week of gestation. The fistula consists of a thin, fragile membranous tract that is closely associated with important structures such as the recurrent laryngeal nerve, and always leads to acute inflammation and surrounding scar tissue. Third and fourth branchial anomalies arising from the pyriform fossa are often indistinguishable [3,4]. PSF has been reported to present at any age, from diagnosis in utero to adulthood. These anomalies can be dangerous in neonates because of rapid enlargement, leading to tracheal compression and respiratory distress [3,5]. In our study, a giant cervical cyst was detected in utero and had rapid enlargement in a few days after birth, but it became smaller than 1 cm in a few weeks after the OK-432 injection.

The diagnosis of PSF is usually established by barium esophagography [6]. An intravenous contrast-enhanced CT has been considered useful for determining the location and extent of the lesion. MRI, ultrasound, and laryngoscopy are essential investigations of choice [7]. Among these modalities, barium esophagography can be repeated without any pain or sedation. However, since barium remains in the PSF for one or two days after esophagography, chemocauterization should be done at least a week after the barium is washed out completely. CT is also a useful diagnostic modality for PSF, showing the extent of inflammation and abscess. Cha et al. reported that the sensitivities of CT and barium esophagography were 100% and 65.9%, respectively [8]. However, gas forming along the sinus tract on CT was 37.1% in the article, indicating that CT is not always enough as a tool for precise diagnosis. A variety of imaging modalities should be used to ensure the diagnosis of PSF.

Since anomalies of the fourth branchial pouch were first described in 1973 [5] and were reported as one of the causes of acute suppurative thyroiditis in 1979 [3], the standard treatment of PSF includes open surgery of the fistula and related hemithyroidectomy. Because adhesion can result from recurrent infections, repetitive incisions, and drainage, identifying the entire fistulous tract during surgery is difficult. Radical dissection may induce adjacent organ injuries such as vocal cord paralysis caused by the recurrence of nerve injury. Despite radical dissection, the complete removal of the fistulous tract is quite difficult, and a remnant fistulous tract can result in the recurrence of inflammation after surgery, which induces a terrible scar on the neck. Nicoucar et al. reported a 15% recurrence rate after open-neck surgical procedures [6]. To avoid these problems, endoscopic cauterization of the internal orifice of the PSF using electrocautery or chemical products was invented. Jordan et al. reported seven cases of electrocauterization through the internal orifice of the PSF in 1998 [9]. Kim et al. subsequently published chemocauterization of the internal orifice of the PSF with TCA in 18 cases in 2000 [1]. The corrosive effect of TCA on the human body is well known, and TCA is generally used clinically for the peeling of superficial lesions. Application of TCA to skin or mucosa induces denaturalization, precipitation, and destruction of the tissue. Using these properties various fistulas, such as tracheoesophageal fistula, congenital fistula from the accessory parotid gland, and small tracheocutaneous fistula in the head and neck, have been treated and reported the feasibility of TCA chemocauterization [10-12]. Since 20% TCA has been frequently used for endoscopic chemocauterization, it has been adopted in our study [4,13,14].

In a systematic review by Derks et al., electrocauterization showed a success rate ranging from 66.7% to 100%, and a cumulative success rate of 77.8% to 100% [15]. Endoscopic cauterization with TCA had a success rate of 77.3% to 83.3% after the first treatment and 87.5% to 91.7% after the second treatment [15]. Both electrocautery and TCA were used to obliterate the internal orifice of the PSF alone [1,7,8,13]. HuDHE was designed by Oshio for both obliterating the entire fistulous duct and keeping TCA overflowing into the hood [16]. The hoods were made from translucent silicon tubes at each hospital because the hoods made by manufacturers were too large to be attached to endoscopes used in children. There are three major advantages for HuDHE over conventional TCA chemocauterization. First, HuDHE is performed not only on the internal orifice of the PSF but also on the entire fistula and can keep TCA inside the hood by pushing the hood against the internal orifice of the PSF (Figure 4), limiting mucosal color change around the PSF. Second, endoscopy can accurately show the detail of mucosal degeneration around the PSF, and almost the entire fistulous duct can be observed by flushing with saline (Figure 5), for both of which the hood provides the optimal field of view. Finally, even if TCA leaks outside the hood and spreads into the larynx or esophagus, causing mucosal erosion, the TCA can be quickly diluted endoscopically with saline.

Interestingly, toothpaste-like secretion was extruded from the PSF during suction and compression before TCA injection in three cases (cases 1, 3, and 6). The secretion appeared to be a waste product that had accumulated inside the PSF over the years but was not visible clearly on any preoperative imaging modalities. It is important to compress the anterior neck and suction the fistulous duct before HuDHE to remove secretion and fill the PSF with TCA completely.

Complications caused by TCA are rare and generally not severe. TCA leaked outside the hood in four children, three (75%) of whom had a transient sore throat. The hood may have made TCA leakage minor. Since the number of TCA injections in each surgery was not recorded in all cases, optimal TCA injection time and volume to prevent TCA leakage are unclear. It is therefore important to confirm that TCA overflows into the hood. None of the children experienced serious complications such as vocal cord paralysis. Park et al. reported two cases of temporary vocal fold immobility due to possibly recurrent laryngeal nerve stimulation after TCA chemocauterization [4]. The vocal cord movement normalized spontaneously within eight weeks in both cases. The depth of permeation of 80% TCA applied with a cotton application to inferior turbinate mucosa for hypertrophic rhinitis is about 120 μm [17]. Although the affected depth after 20% TCA exposure in PSF has not been described yet, it may not degeneralize the recurrent laryngeal nerve permanently even after the mucosa of the PFS is branched. No serious permanent complications after TCA chemocauterization have been reported. It is also necessary to care about how deep the tube is inserted into the fistulous duct. If the tube accidentally pierces the bottom of the PSF, the mucosa may rupture, and TCA may overflow outside the PSF.

There are four major limitations to HuDHE. First, there are no guidelines about how many times cauterization can be attempted without conversion to an external approach. Cha et al. performed a third chemocauterization with TCA in one patient [8]. Eventually, the patient underwent total excision because of recurrence after the third chemocauterization. Second, the closure of the entire fistulous duct is yet to be reported. It seems to be difficult to confirm the closure by using any existing imaging modalities. Closure of the internal orifice of the PSF can be confirmed by either endoscopy or esophagography. Third, hoods attached to the tip of the endoscopes have to be made at each facility. If the commercial product was available in HuDHE, it would contribute to standardizing the procedure of HuDHE. Finally, there are not enough number of treatments, and the follow-up period is short in this study. In future studies, a randomized trial could be conducted to confirm the effectiveness of HuDHE. However, because of the low prevalence of PSF, it would be difficult to conduct such a study.

## Conclusions

HuDHE has the possibility to treat not only the internal orifice of the PSF but also the entire fistulous duct for definitive cure. HuDHE may be a less invasive, safe, and effective treatment option for children with PSF.
